# Longitudinal Study of Cytokine Expression, Lipid Profile and Neuronal Growth Factors in Human Breast Milk from Term and Preterm Deliveries

**DOI:** 10.3390/nu7105415

**Published:** 2015-10-19

**Authors:** Maria Carmen Collado, Marina Santaella, Laia Mira-Pascual, Elena Martínez-Arias, Parisá Khodayar-Pardo, Gaspar Ros, Cecilia Martínez-Costa

**Affiliations:** 1 Department of Biotechnology, Institute of Agrochemistry and Food Technology-Spanish National Research Council (IATA-CSIC), Paterna 46980, Valencia, Spain; laimipas@alumni.uv.es; 2 Department of Food Technology, Food Science and Nutrition, Faculty of Veterinary Sciences, Regional Campus of International Excellence “Campus Mare Nostrum”, University of Murcia, Espinardo 30071, Murcia, Spain; marinasp@um.es (M.S.); gros@um.es (G.R.); 3 Pediatric Gastroenterology and Nutrition Section, Department of Pediatrics, University Hospital of Valencia, University of Valencia, Valencia 46010, Spain; emara_87@hotmail.com (E.M.-A.); parisa.khodayar.pardo@gmail.com (P.K.-P.)

**Keywords:** human milk, breast milk, breastfeeding, fatty acids, cytokines, growth factors, neurotrophic factor, polyunsaturated fatty acids, docosahexaenoic acid

## Abstract

Breast milk (BM) is considered as a reference for infant nutrition. The role of bioactive components, such as cytokines, hormones, growth factors (GFs) and fatty acids (FAs) is poorly known, but they might be implicated in immune response development. The aim of this study was to identify the lipid profile and the spectrum of cytokines and neuronal GF in BM samples and analyse the influence of gestational age and lactation time on these components. This study used a longitudinal prospective method for the characterization of cytokines, FAs and GFs global profiles in 120 BM samples from 40 healthy mothers (20 preterm and 20 term) collected as colostrum, transitional and mature milk. The cytokines were analysed by protein array (Ray Bio® Human Cytokine Array G6. Ray Biotech, Inc. Norcross, GA, USA) and the FAs were analysed by gas chromatography. The FA profile was similar between the term and the preterm BM samples. Omega-3-α-linoleic and docosahexaenoic acid (DHA) and omega-6-linoleic acid were the most abundant in the term and preterm samples during lactation. Omega-3 ETA and omega-3 EPA we observed exclusively in the preterm samples. The cytokine profile showed a different trend based on gestational age. A significantly higher expression of neurotrophic factors was found in the mature preterm milk samples as compared to the mature term samples. Our study is the first to identify the influence and interactions of perinatal factors on cytokine, GFs and FAs in human milk.

## 1. Introduction

Several studies have found that maternal milk is the optimal source of nutrients and an unmatched supply of essential protective biocomponents for human infants, particularly during the first months of life. These bioactive components include oligosaccharides, hormones, enzymes, immunoglobulins (mainly secretory, Immunoglobulin A (IgA), lactoferrin (Lf), growth factors (GF’s), cytokines, anti-inflammatory agents and microbial factors, which are poorly understood and which are probably implicated in immune response development [1,2,3]. Cytokines are pluripotent polypeptides that act in autocrine/paracrine modes by binding to specific cellular receptors [4]. These proteins regulate the inflammatory responses stimulated by an antigenic challenge and contribute to the development and maturation of the immune system [5]. Numerous cytokines as interleukin, interferon, macrophage-colony stimulating factor, Tumor Necrosis Factor-α (TNF-α, granulocyte-colony stimulating factor (G-CSF), *etc.* have been identified in breast milk (BM) suggesting that they can be interconnected in the control of the inflammation and infection response [4,6,7,8,9,10]. Other molecules, such as GFs, can contribute to the development of several structures, including neuronal components, which are extremely important for the development of the enteric and central nervous systems [8,11,12].

Regarding nutritional components, human milk is considered to be the source of nutrient balance for a term infant. Lipids constitute the highest macronutrient components of breast milk and includes specially long-chain polyunsaturated fatty acids (LC-PUFAs), which not only has a nutritional function but also metabolic functionality, that are involved in brain development and are one of the main components of the neuronal membrane. LC-PUFAs consist, among other things, of docosahexaenoic acid (DHA) (an omega-3 FA), and arachidonic (AA) (an omega-6 FA), which are influenced by the mother’s diet and other environmental factors [13] and related to brain development, like in a cohort of pregnant women, it has been demonstrated that maternal supplementation of very-long-chain omega-3 PUFAs during pregnancy and lactation is favourably for later mental development in children [14], and other investigation supported the influence of omega-3 on cognitive development [15,16,17]. However, foetal accretion of DHA occurs from the last trimester of gestation that increase the problem into immature infant (usually weighing less than 2500 grams at birth and not physiologically well developed) has substantial difficulty synthesizing DHA from elongation and desaturation of its FA precursors [13], and that lead the preterm births (born before the thirty-seventh completed week of gestation) at risk of DHA deficiency [18]. Hypothetically, all of these components could act synergistically in neural development and in response against inflammation and infection. Several studies have investigated cytokines and growth factors in human milk, however, scarce data on the relationship between milk compounds including immunological and FA profiles, are available. Based on the previous information of the development of human milk from immature to mature, and the relationship between some bioactive and key nutrients of mother milk with immune system, the aim of this study was to study the impact of gestational age on the evolution during lactation of the lipid profile (FA Profile), and the spectrum of cytokines and neuronal growth factors (GFs) and also, on their interactions.

## 2. Material & Methods

### 2.1. Subjects and Design

A longitudinal prospective study of the characterization of FA profile, cytokines and GFs in 120 breast milk samples from 40 healthy mothers was conducted. The mothers were recruited after delivery at the Maternity Ward of the Hospital Clínico Universitario de Valencia (Spain) between 2008 and 2014. The study was approved by the Ethics Committee of the Hospital and the Bioethics Subcommittee of Consejo Superior de Investigaciones Científicas (CSIC). The study complied with the Declaration of Helsinki, as reviewed in 2000.

To assess the influence of perinatal factors, the breast milk samples were divided into two groups depending on gestational age: 20 term gestations (infants born at or after 37 weeks of gestation) and 20 preterm gestations (infants born before 37 weeks of gestation) (Table 1). Samples were collected within the first month of exclusive breastfeeding and categorized into three subgroups based on lactation stage as follows: colostrum (1st–6th day postpartum), transitional (7th–15th day postpartum) and mature milk (from the 16th day onwards).

**Table 1:** Clinical characteristics of mother-infant pair included in the study.

	Term Gestations (*n* = 20)	Preterm Gestations (*n* = 20)
**Maternal age (year)**	30 (25.0–32.8)	32.1 (27.2–33.7)
**Length of pregnancy (week)**	39.2 (38.0–40.0)	29 (27–315)
**Mode of delivery**		
Vaginal (%)	10 (50%)	6 (30%)
C-section (%)	10 (50%)	14 (70%)
**Birth weight (g)**	3200 (3000–3650)	2850 (2500–3000)

Data are shown as median and interquartile range (IQR).

### 2.2. Breast Milk Samples

Before the sample collection, mothers were given written instructions for the standardized collection of samples each morning. They washed their hands with soap and their breasts were cleaned with a swab of 0.5% chlorhexidine solution to reduce the amount of bacteria residing on the skin. The milk sample collection was carried out with a sterile automatic breast milk pumper with a vacuum regulator (Medela Symphony®, Barr, Switzerland), polystyrene suction funnels and screw-top bottles adapted to suction funnels for direct collection of milk. The bottles and the suction funnels were autoclaved before use. The milk samples suctioned with the pumper were collected in bottles (25 mL approximately) and immediately aliquoted in 5 mL tubes, using sterile material, frozen and stored at −80 °C for later analysis [19].

The fatty layer and cellular elements of the breast milk were removed by two subsequent centrifugations at 4,000 rpm for 20 min at 4 °C, and stored at −80 °C for FAs analysis. The remaining whey milk was centrifuged at 14,000 rpm for 20 min at 4 °C, and the supernatant was used for the protein array analysis.

### 2.3. Fatty Acid Analysis

The fatty layer containing the FAs was dissolved in hexane and the fatty esters were methylated by 2N potassium hydroxide in methanol. The separation and quantification of the different fatty acids were done with gas liquid chromatography using an Agilent Technologies 7890A device, (Agilent Technologies, Palo Alto, CA, USA) equipped with a flame ionisation detector (FID) operated with a split ratio of 20:1. The column was a DB-23 (60 m, 0.25 mm *i.d*., 0.25 μm coating thickness (Agilent Technologies). The injector and detector temperatures were held constant at 250 °C and 280 °C, respectively. The oven temperature was programmed at 50 °C for 1 min and ramped at 25 °C/min to 175 °C, followed by further ramping at 4 °C/min to 220 °C where the temperature was held constant at 220 °C for 18 min. The carrier gas, helium, was held at a constant pressure of 230 kPa. Chromatographic air and hydrogen (400 mL/min and 35 mL/min, respectively) were supplied to the FID. The methyl esters were identified by comparing the retention time of the unknowns with those of the known FA methyl ester (FAME) standards (Supelco 37 component FAME Mix and FAME marine source, both from Supelco, St. Louis, MO, USA). The relative proportion of each FA in the flesh was reported as a percentage of the total FAME present in the injected sample. The sum of the saturated FA (SFA), the monounsaturated FA (MUFA), the polyunsaturated FA (PUFA) and the highly unsaturated FA, both the *n*-3 series and the *n*-6 series and the ratio between *n*-3 FA and *n*-6 FA, were also calculated.

### 2.4. Protein Array Analysis

Protein concentrations were measured using the Bradford Protein Assay (Bio-Rad) and samples were normalized at 30 μg of protein for the analysis. The biological samples were then pooled, including different biological samples in each pool (a total of 12 pools including 10 biological samples were analysed). All of the pooled sample measurements were performed in duplicate. The cytokine profiles were analysed with a semiquantitative human cytokine antibody array that detects 60 proteins (Table A1) in one experiment (RayBio Human Cytokine Antibody Array G series VI; Raybiotech Inc., Norcross GA, USA). The array glass slides were washed, incubated with a biotin-conjugated anti-cytokine mix for 2 h, washed again, and developed for 2 h with Cy3-conjugated streptavidin. The signals were scanned with a GenePix 4000B scanner (Axon Instruments, GenePix version 5.0, San Francisco, CA, USA) and analysed with the Raybiotech analysis tool (Raybiotech Inc., Norcross, GA, USA), a data analysis program based on Microsoft Excel technology specifically designed for the Raybiotech Antibody Array G Series. Signals were normalized using the internal, positive and negative controls included in the array. Mean fold-change values obtained in three independently performed experiments were calculated and used to rank the expression of cytokines. Any >1.5-fold increase or ≤0.65-fold decrease in signal intensity for a single analyte between samples or groups may be considered to be a measurable and significant difference in expression, provided that both sets of signals are well above background (Mean background + 2 standard deviations, accuracy 95%).

### 2.5. Statistical Analysis and Bioinformatics

Statistical analysis for the validation experiments (means, medians, range, standard deviations, significance of group differences and linear regression) were evaluated using IBM SPSS software (release 19.0, SPSS, Inc., Chicago, IL, USA). Between-group comparisons were performed with Student’s *t* test. *p* values < 0.05 were considered significant. Correlation of the parameters was analysed with Pearson’s correlation analysis. Statistical significance was defined as a two-sided *p*-value < 0.05. Principal Component Analysis (PCA) was conducted using SIMCA 14.0 (Umetrics, Malmö, Sweden).

## 3. Results

### 3.1. Fatty Acids Analysis

**Figure 1:**
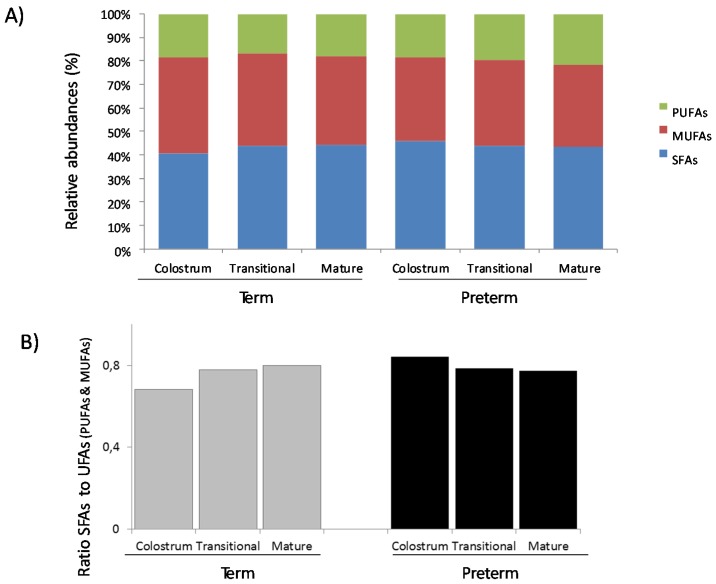
Relative abundances (%) of fatty acids (FAs) (Panel A) and ratio of saturated (SFAs) to unsaturated-UFAs (PUFAs and MUFAs) fatty acids (Panel B) present in breast milk samples according to gestational age and lactation stage.

**Figure 2:**
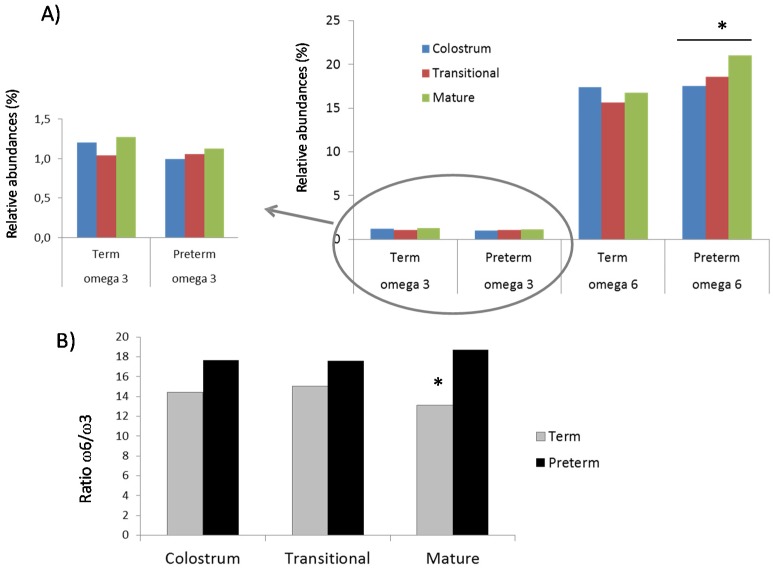
Relative abundances of unsaturated fatty acids (Panel A) and ratio omega-6 to omega-3 (Panel B) in breast milk samples. The ratio omega-6 to omega-3 was significantly different (* *p*-value < 0.05) between term and preterm samples.

The relative abundances of FAs present in BM samples (SFAs, MUFAs and PUFAs) were similar between groups (Figure 1A) almost the same for SFAs and MUFAs (around 40% for colostrum, transitional and mature as well) and half for PUFAs (around 20%). The ratio of SFAs (Figure 1B) to unsaturated FAs (MUFAs and PUFAs) showed an increased tendency in human milk term samples compared to the opposite trend observed in human milk preterm samples during lactation. Levels of omega FAs showed a different trend in term and preterm human milk samples and along the three periods of sampling (Figure 2A). So omega-3 FAs increased slightly but during transition period, and omega-6 FAs decreased in term samples compared to preterm samples, were increase was and with statistical significance in omega-6 FAs. The ratio of the percentages of omega-6 to omega-3 (Figure 2B) was higher in preterm than in term samples and it was significant in mature milk.

**Figure 3:**
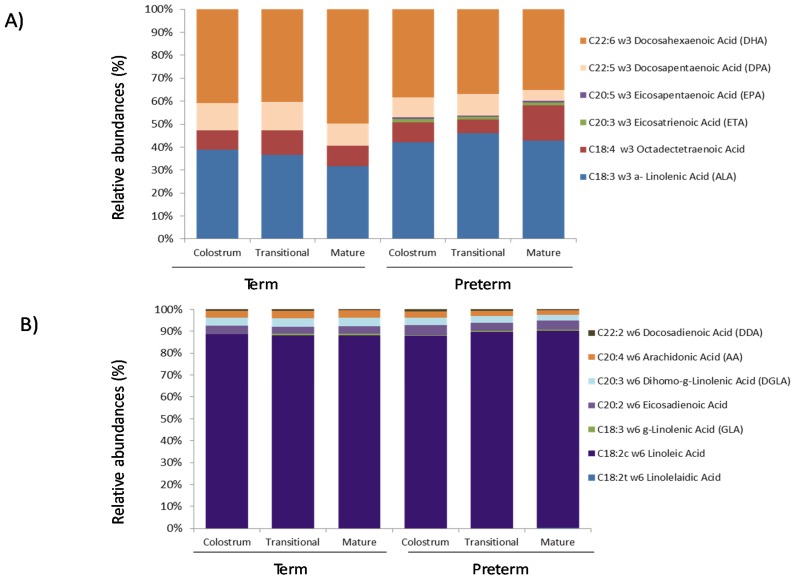
Relative abundances (%) of different PUFAs omega 3 family components (Panel A) and omega-6 family components (Panel B) present in breast milk samples analysed by gas chromatography and grouped according to gestational age and lactation stage.

The relative abundances of specific PUFAs omega 3 and omega-6 showed different trends between term and preterm during lactation (Figure 3). Omega-3 C18:3 *n*-3 ALA and C22:6 *n*-3 DHA (Figure 3A) and omega 6 C18:2c *n*-6 Linoleic acids (Figure 3B) were the most abundant in term and preterm samples during lactation. Remarkable is the finding that C20:3 *n*-3 ETA (Eicosatrienoic acid) and C20:5 *n*-3 EPA (Eicosapentaenoic acid) FAs appeared exclusively in preterm samples.

**Figure 4:**
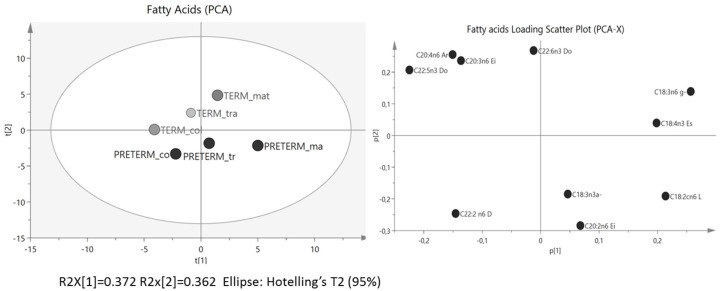
Principal Component Analysis (PCA) and Loading Scatter Plot of the fatty acid profile (*n* = 40 fatty acids determined) obtained by gas liquid chromatography. By comparing the score and loading plot, the relationships between fatty acids and samples (term and preterm during lactation) could be identified.

The FAs Principal Component Analysis (PCA) (Figure 4) showed a differential FA behaviour in the term and preterm samples, where they follow a parallelism in colostrums and transitional (difficult to establish with only two points), but in mature human milk samples, they clearly separate themselves, mainly in preterm mature milk. The principal component 1 (PC1) explained the differential traits in 37.2% and FA profile evolved a long PC1 from colostrum to mature milk. In addition, preterm samples were grouped in the lower part.

### 3.2. Protein Array Analysis

**Figure 5:**
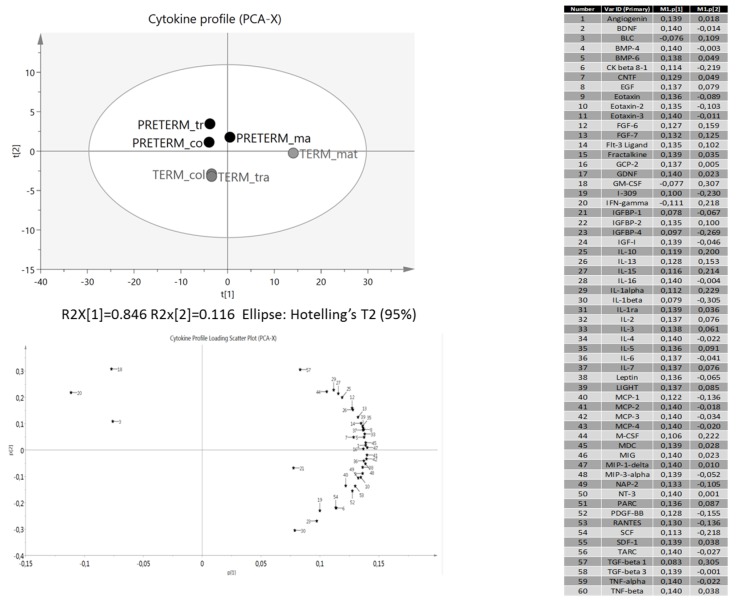
Principal Component Analysis (PCA) and Loading Scatter Plot of cytokine profile (*n* = 60 cytokines) obtained by protein arrays from milk samples. By comparing the score and loading plot, the relationships between cytokine profiles and samples (term and preterm during lactation) could be identified.

**Figure 6:**
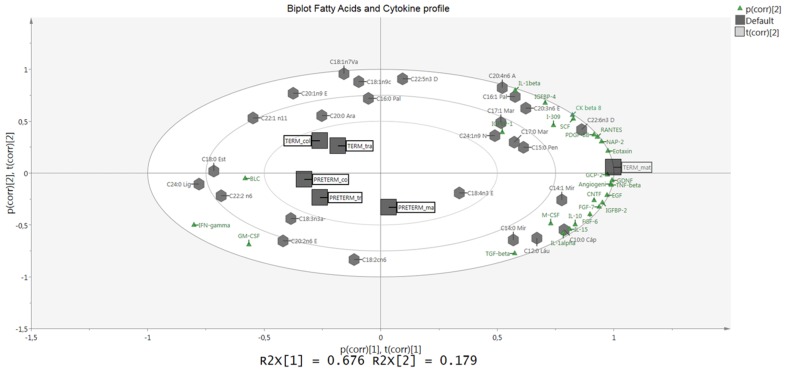
Biplot PCA model representing the correlation structure of the dataset in a two-dimensional space of the fatty acid profile (*n* = 40 fatty acids determined) obtained by gas liquid chromatography and cytokine profile (*n* = 60 cytokines) obtained by protein arrays from milk samples.

Specific cytokine profiles were found in each analyzed group (Table 2). We observed higher abundance of Angiogenin; Epidermal Growth Factors (EGF); Interleukin-6 (IL-6); B-Lymphocyte Chemoattractant (BLC); Insulin-like Growth Factor-Binding Protein 1 and 2 IGFBP-1 & IGFBP-2 and Monocyte Chemotactic Protein 1 (MCP-1) in the term group while EGF; IGFBP-2; BLC; Angiogenin; IL-6 and Macrophage-Colony Stimulating Factor (M-CSF) were higher in the preterm group. The PCA analysis showed a differential cytokine profile between term and preterm samples (Figure 5). The PC1 explained the differential traits in 84.6% and preterm samples are grouped in same area while colostrum and transitional term samples are clustered. We could also detect that a higher PC1 was related to lactational stage in preterm. The differential cytokines between term and preterm samples were BLC; Interferon gamma (IFNγ); Granulocyte Macrophage-Colony Stimulating Factor (GM-CSF), which were related to preterm samples, and Transforming Growth Factor-β 1 (TGF-β1); IGFBP-1 and Interleukin-1 (IL1-β) related to term sample. We detected significantly higher expression of neurotrophic factors in mature preterm milk compared to mature term samples (Table 2), no differences were found between colostrum and transitional milk. We also found that the other growth factors and cytokines were significantly higher in preterm than in term samples. In addition, a specific cytokine and FA profile was found to be related to the groups analyzed (Figure 6). The biplot showed a direct association with preterm samples and also with term.

**Table 2:** Fold change table comparing cytokine profile according to lactation stage and gestational age.

A: Fold change table comparing cytokine profile according to lactation stage			
	Colostrum *vs*. Transitional Milk	Colostrum *vs*. Mature Milk	Transitional *vs*. Mature Milk
**Neurtophic factors**			
Brain-derived neurotrophic factor (BDNF)	1.00	0.36 *	0.36 *
Ciliary neurotrophic factor (CNTF)	0.96	0.34 *	0.35 *
Glial cell-derived neurotrophic factor (GDNF)	0.94	0.14 *	0.15 *
Neurotrophin-3	0.91	0.23 *	0.25 *
**Other growth factors**			
Angiogenin	0.99	0.20 *	0.21 *
Bone morphogenetic protein 4	0.85	0.07 *	0.08 *
Bone morphogenetic protein 6	1.07	0.46 *	0.43 *
Epidermal growth factor	0.84	0.13 *	0.16 *
Fibroblast growth factor 6	0.65 *	0.20 *	0.30 *
Fibroblast growth factor 7	0.83	0.38 *	0.46 *
Fms-related tyrosine kinase-3 ligand	0.88	0.38 *	0.43 *
Insulin-like growth factor-binding protein 1	1.44	1.05	0.73 *
Insulin-like growth factor-1	1.03	0.37 *	0.35 *
Platelet-derived growth factor	1.08	0.58 *	0.54 *
**Cytokines**			
CK β 8-1	1.13	0.62 *	0.55 *
IFN-gamma	0.90	1.81 *	2.01 *
IL-1β	1.06	0.88	0.83
IL-1α	0.83	0.46 *	0.55 *
IL-2	0.91	0.44 *	0.48 *
IL-3	1.00	0.29 *	0.29 *
IL-4	1.07	0.35 *	0.33 *
IL-5	0.89	0.33 *	0.37 *
IL-6	0.95	0.45 *	0.47 *
IL-7	0.86	0.33 *	0.38 *
IL-10	0.76	0.35 *	0.45 *
IL-13	0.66	0.26 *	0.39 *
IL-15	0.78	0.41 *	0.53 *
Monokine induced by gamma interferon	0.99	0.60 *	0.61 *
Neutrophil-activating protein-2	1.01	0.50 *	0.49 *
Thymus and activation-regulated chemokine	0.98	0.48 *	0.49 *
Transforming growth factor-β 3	0.95	0.52 *	0.55 *
Tumor necrosis factor-α	0.95	0.45 *	0.47 *
Tumor necrosis factor-β	0.95	0.20 *	0.21 *
**Chemoattractant cytokines**			
Eotaxin	1.14	0.30 *	0.27 *
Fractalkine	0.84	0.19 *	0.23 *
Granulocyte chemotactic protein 2	0.95	0.47 *	0.49 *
Granulocyte-macrophage colony-stimulating factor	0.88	1.41	1.61 *
I-309	0.89	0.56 *	0.63 *
Monocyte chemoattractant protein-1	1.05	0.72	0.69
Monocyte chemoattractant protein-2	1.03	0.34 *	0.33 *
Monocyte chemoattractant protein-3	0.91	0.40 *	0.44 *
Monocyte chemoattractant protein-4	0.99	0.38 *	0.38 *
Macrophage colony-stimulating factor	0.95	0.45 *	0.47 *
Human macrophage-derived chemokine	1.07	0.39 *	0.37 *
MIP-1-delta	0.82	0.12 *	0.15 *
MIP-3-α	1.26	0.24 *	0.19 *
Pulmonary and activation-regulated Cytokine (PARC)	0.83	0.18 *	0.22 *
Stromal cell-derived factor-1	0.97	0.31 *	0.32 *
**B: Fold change table comparing cytokine profile according to gestational age**			
	**Preterm *vs.* Term colostrum**	**Preterm *vs.* Term transitional**	**Preterm *vs.* Term mature**
**Neurtophic factors**			
Brain-derived neurotrophic factor (BDNF)	0.89	0.90	2.14 *
Ciliary neurotrophic factor (CNTF)	0.96	0.96	2.04 *
Glial cell-derived neurotrophic factor (GDNF)	1.39	1.28	5.06 *
Neurotrophin-3	1.10	0.98	3.60 *
**Other growth factors**			
Angiogenin	0.74	0.89	5.23 *
Bone morphogenetic protein 4	1.00	0.48 *	12.80 *
Bone morphogenetic protein 6	1.12	1.12	1.83 *
Epidermal growth factor	0.94	6.61 *	2.78 *
Fibroblast growth factor 6	1.14	1.10	1.54
Fibroblast growth factor 7	1.01	1.85 *	1.40
Fms-related tyrosine kinase-3 ligand	1.13	1.62 *	1.34
Insulin-like growth factor-binding protein 1	0.70	1.27	3.80 *
Insulin-like growth factor-1	0.88	0.76	2.43 *
Platelet-derived growth factor	1.11	0.45 *	2.68 *
**Cytokines**			
CK β 8-1	1.07	0.27 *	3.26 *
IFN-gamma	0.98	2.07 *	0.63 *
IL-1β	1.18	0.40 *	1.70 *
IL-1α	0.95	2.54 *	1.24
IL-2	0.87	1.44	1.59 *
IL-3	1.13	1.46	2.15 *
IL-4	0.90	0.86	2.39 *
IL-5	0.84	1.95 *	1.52 *
IL-6	0.71	0.97	1.97 *
IL-7	0.78	1.40	1.81 *
IL-10	2.87 *	4.08 *	2.09 *
IL-13	1.99 *	3.13 *	2.20 *
IL-15	1.96 *	2.57 *	1.21
Monokine induced by gamma interferon	0.90	1.07	1.47
Neutrophil-activating protein-2	0.89	0.59 *	3.89 *
Thymus and activation-regulated chemokine	1.03	0.90	1.67 *
Transforming growth factor-β 3	0.97	1.11	1.62 *
Tumor necrosis factor-α	0.88	0.85	1.79 *
Tumor necrosis factor-β	0.99	1.46	2.84 *
**Chemoattractant cytokines**			
Eotaxin	0.97	0.26 *	5.78 *
Fractalkine	1.54 *	1.27	2.71 *
Granulocyte chemotactic protein 2	1.80 *	0.80	1.97 *
Granulocyte-macrophage colony-stimulating factor	0.99	2.53 *	0.87
I-309	1.37	0.20 *	2.21 *
Monocyte chemoattractant protein -1	1.07	0.63 *	5.97 *
Monocyte chemoattractant protein -2	0.87	0.79	2.02 *
Monocyte chemoattractant protein -3	0.80	0.78	1.74 *
Monocyte chemoattractant protein -4	0.97	0.86	2.79 *
Macrophage colony-stimulating factor	0.56 *	5.41 *	5.61 *
Human macrophage-derived chemokine	0.81	1.36	2.81 *
MIP-1-delta	0.66	0.91	1.92 *
MIP-3-α	0.67	0.40 *	6.88 *
Pulmonary and activation-regulated Cytokine (PARC)	1.14	3.02 *	3.02 *
Stromal cell-derived factor-1	0.95	1.20	1.56 *

Any >1.5-fold increase or ≤0.65-fold decrease in signal intensity for a single analyte between samples or groups may be considered a measurable and significant difference in protein expression (* *p* value < 0.05); IFN-gamma, interferon-gamma; IL, interleukin; MIP, macrophage inflammatory protein.

## 4. Discussion

Breastfeeding serves as a source of optimal nutrition and bioactive factors for infant development, especially when maternal nutrition is optimal. Human milk fat content and omega-3 FA (DHA) are crucial for infant neurodevelopment [8,11,12,13,14,15,16,17]. Omega-3 FAs have been proven to contribute to neurophysiological development and disease prevention in infants and children [20]. Omega-3 DHA and omega-6 arachidonic acid (AA) are both concentrated in the infant brain early in life, reaching approximately 50%–60% of brain dry weight in adults [14,21].

In agreement with previous research, we found higher variability in the FAs profile in term samples [22]. Compared to the findings of other researches, our data showed higher concentrations of SFA C12:0 lauric acid and C14:0 myristic acid in colostrum and similar levels of C16:0 palmitic acid and C18:0 stearic acid during the three stages of lactation in term samples [22]. Regarding gestational age, similar concentrations of FA were reported in the majority of term and preterm samples. However, SFA levels were slightly higher in colostrum preterm samples than term samples. This observation was reflected in the ratio of saturated FAs (SFAs) to unsaturated FAs (PUFAs and MUFAs): the ratio increased in term samples but decreased in preterm samples (Figure 1B).

Among MUFAs, the C18:1 oleic acid levels in term samples were similar to those found in previous research [22], while those in preterm samples were lower. In the main result for PUFAs, C18:2 *n*-6 linoleic acid levels were more than twice the reported levels for term and preterm samples, reaching the highest levels in mature milk in term samples [22]. However, our data agree with the percentages of C18:2 *n*-6 linoleic acid found during the three lactation stages in term milk samples drawn from a Spanish population [23]. This observation supports the claim that maternal diet and geographical location might affect PUFA concentrations.

Regarding omega-3 FA, we detected higher levels in all term samples than preterm samples. The levels of C18:3 *n*-3 -linolenic acid in mature milk samples were similar to those previously reported [22]. In addition, a significant increase in omega-6 levels was shown in the preterm group during lactation, resulting in a higher omega-6 to omega-3 ratio in the preterm samples than the observed samples. However, the increase was significant only in mature milk. The omega-6 to omega-3 ratio in term samples was comparable to that in previous data from country-specific populations [23].

Our results show similar concentrations of DHA in term samples (0.49%–0.64% during lactation) and preterm samples (0.38%–0.40%). These levels are higher than in other studies [13] but in the same range as those reported in our country, Spain [23]. In agreement with previous data [24], we report high variability in DHA levels in breast milk samples. A recent study conducted with Hungarian women found lower DHA levels in colostrum than our results, with a significant decrease during lactation [25]. The likely explanation of these differences is that maternal diet can change the composition but not the total amount of FA [22,26]. A meta-analysis of 65 studies on mature human milk involving 2474 women reported an average DHA level of 0.32%± 0.22% and AA level of 0.47% ± 0.13% [13]. The DHA levels exhibited great variability by country, with the highest concentrations found in the Canadian Arctic, Japan, Dominican Republic, Philippines and Congo. Studies from Spain also found greater DHA concentrations when fish consumption was high [13]. Omega-3 FA DHA levels have been compared in breast milk samples from a rural area (Amazonian Bolivia) and the United States [27]. The same study reported omega-6 to omega-3 ratios of approximately 4:1, while this ratio varied from 10:1 to as high as 20:1 in industrialized diets [27]. High levels of omega-6 have been linked to increased risk of obesity, inflammation and cardiovascular disease and have been reported to interfere with the synthesis of DHA and other omega-3 fatty acids [27]. As also shown in our data, other researchers reported no differences in arachidonic acid and DHA levels in between term and preterm milk samples [28].

We described specific protein profiles in milk samples according to gestational age and lactation stage (Figure 5 and Table 2). These protein profiles might have different main biological activities, and some display multiple functional properties, including anti-inflammatory activities and chemoattractant functions. The majority of cytokines was present in colostrum and decreased during lactation. However, IFN-γ and M-CSF remained elevated during lactation in term samples. IFN-gamma is considered a crucial immune modulator in response to intracellular pathogens, and GM-CSF is an important hematopoietic growth factor, which has received attention in recent research for its application as an immune adjuvant due to its ability to increase dendritic cell maturation and function and macrophage activity [29]. We also found cytokines with chemoattracting activity in colostrum and milk samples, which, as reported, include PARC/CCL18, MIP-3α/CCL20 (mainly for lymphocytes and immature dendritic cells), MIP-1β/CCL4 (mainly for NK cells and macrophages) and B lymphocyte chemoattractant/CXCL13 (mainly for naive B lymphocytes) [8]. These activities which attract various types of cells could benefit the proper development and priming of the intestinal lymphocytes, which might protect newborns against several diseases immediately after birth and later in life [30].

In preterm infants, most cytokines increased during lactation and were significantly higher than in term samples. This trend could be related to the higher protein levels in preterm milk than term milk during the first month of lactation and could function as a protective factor against infection and inflammation. Similar patterns have been found: trophic peptides as an epidermal growth factor (EGF) and a transforming growth factor-α (TGF-α) were elevated in the milk of preterm infants [31]. Levels were significantly higher in extremely preterm (23–27 weeks) samples than older preterm and full-term samples [31].

In agreement with previous research [8], we found specific growth factors, such as fibroblast growth factors (FGF-6 and FGF-7), which might contribute to the development of gut structure and function, including epithelial barrier maturation. Other growth factors, such as platelet-derived growth factor (PDGF-BB), might mostly influence angiogenesis. Interestingly, we also found neurotrophic factors, such as brain-derived neurotrophic factor (BDNF), glial cell line-derived neurotrophic factor (GDNF), and neurotropin-3 (NT-3). BDNF and GDNF induce the growth and development of the central nervous system and are required for the survival of some primary sensory neurons during foetal development [32]. Neurotropin-3 acts simultaneously with GDNF and is considered important to the normal development of the infant enteric nervous system [12]. A recent report confirmed our finding of elevated levels of the transforming growth factor-β (TGF-β), which decreased during lactation. Additionally, the recent report found that infants with necrotizing enterocolitis showed decreased levels of this cytokine, suggesting another benefit of breastfeeding [33].

In summary, lipid concentration provides a major nutrient in breast milk and includes long-chain polyunsaturated fatty acids (LCPUFA), which are involved in brain development and are one of the main components of the neuronal membrane. Researchers have reported higher intellectual abilities in breastfed infants than formula-fed infants, suggesting that omega-3 FAs have a role in brain development [11,15,16,17]. An association among cognition, DHA and the neurotrophic factor BDNF in human milk has been suggested [11]. Experimental studies have shown the protective effect of GDNF and CNTF in enteric neuronal survival and suggested that breast milk can promote neuronal differentiation in rats [34]. The potential interactions between neurotrophic factors and specific FAs could be relevant to the development of the nervous system. Accordingly, our results found relationships of the neurotrophic factors CNTF and GDNF with omega-3 EPA and ETA (*p* = 0.019 and 0.088, respectively).

## 5. Conclusions

In conclusion, complex interactions between FA and protein profiles are relevant to infant development. All of these findings support the crucial interest in human milk for infant development, especially in infants born preterm. Considering that the preterm infant is frequently fed with banked human-donor milk, it is important that, as has recently been reported, Holder pasteurization preserves many growth factors [10]. Further research on milk bioactive-factor interactions is needed to understand the biological role of breast milk and its impact on infant development and health.

## Appendix

**Table A1:** The cytokine profiles were analysed with a semiquantitative human cytokine antibody array that detects 60 proteins..

RayBio® Human Cytokine Antibody G Series VI													
POS 1	POS 2	POS 3	NEG	NEG	Angiogenin	BDNF	BLC	BMP-4	BMP-6	CK β 8-1	CNTF	EGF	Eotaxin
Eotaxin-2	Eotaxin-3	FGF-6	FGF-7	Fit-3 Ligand	Fractalkine	GCP-2	GDNF	GM-CSF	I-309	IFN-gamma	IGFBP-1	IGFBP-2	IGFBP-4
IGF-I	IL-10	IL-13	IL-15	IL-16	IL-1α	IL-1β	IL-1ra	IL-2	IL-3	IL-4	IL-5	IL-6	IL-7
Leptin	LIGHT	MCP-1	MCP-2	MCP-3	MCP-4	M-CSF	MDC	MIG	MIP-1delta	MIP-3α	NAP-2	NT-3	PARC
PDGF-BB	RANTES	SCF	SDF-1	TARC	TGF-β1	TGF-β3	TNF-α	TNF-β	IC1	IC2	IC3	NEG	NEG

MCP, monocyte chemotactic protein; IFN-gamma, interferon-γ; IL, interleukin; IGF, insulin-like growth factor; PDGF-BB, platelet derived growth factor-BB; FGF, fibroblast growth factor; SCF, skp1-cullin-F-box ligase; SDF, stromal-derived factor; TGF, transforming growth factor; GCP, granulocyte chemotactic protein; M-CSF, macrophage colony stimulating factor; GDNF, glial cell line–derived neurotrophic factor; TNF, tumor necrosis factor; MIP, macrophage inflammatory protein; IGFBP, insulin-like growth factor binding protein; nap, neutrophil-activating peptide; NT, neurotrophin; BDNF, brain-derived neurotrophic factor; BLC, B-lymphocyte chemoattractant; BMP, bone morphogenetic protein; CNTF, ciliary neurotrophic factor; EGF, epidermal growth factor.
